# Synthesis, Characterization, and Applications of Nanomaterials for Energy Conversion and Storage

**DOI:** 10.3390/molecules28217383

**Published:** 2023-11-01

**Authors:** Jin Jia, Yucheng Lan

**Affiliations:** 1 Key Laboratory of Spin Electron and Nanomaterials of Anhui Higher Education Institutes, Suzhou University, Suzhou 234000, China; 2 Department of Physics and Engineering Physics, Morgan State University, Baltimore, MD 21251, USA

Ever since the commencement of the Industrial Revolution in Great Britain in the mid-18th century, the annual global energy consumption from various fossil fuels, encompassing wood, coal, natural gas, and petroleum, has demonstrated an exponential surge over the past four centuries [1,2]. The finite fossil fuel resources on our planet are diminishing rapidly, and are projected to be completely depleted by the end of this century if the current rate of consumption continues unabated [3,4]. Moreover, the extensive deployment of fossil fuels has led to the release of substantial quantities of greenhouse gases into the Earth’s atmosphere. In 2022 alone, a staggering 36.8 gigatons (Gt) of carbon dioxide (CO_2_) were discharged [5]. The repercussions of these gas emissions are far-reaching and profound, causing significant environmental disturbances and posing grave threats to climate stability and human welfare. In addition, the usage of fossil fuels has resulted in the production of pollutants, including sulfur dioxide (SO_2_), nitrogen oxide (NO*_x_*), ammonia (NH_3_), volatile organic compound (VOC) gases, and heavy metal compounds, which have emerged as global concerns regarding air quality [6]. Such pollution is linked with an escalating risk to human health and ecosystems. Alarmingly, there have been instances of issues such as acid rain, and the number of cancer cases has surged compared to figures from four decades ago, indicating a concerning health impact, particularly for individuals residing in regions with high pollution levels [7]. Therefore, it is of paramount importance to curb the usage of fossil fuels in order to protect the environment and preserve human health. To meet the ambitious target outlined by the Paris Agreement, which aims to restrict global warming to no more than 1.5 degrees Celsius above pre-industrial levels, a 45% reduction in greenhouse gas emissions by 2030 and the realization of net zero emissions by 2050 are required, as specified by the United Nations’ Intergovernmental Panel on Climate Change (IPCC) [5].

In an effort to diminish our dependency on fossil fuels and address the escalating energy demands of modern human activities, a concerted effort has been made to explore various sources of renewable energy. These sources encompass solar energy, wind power, hydroelectricity, geothermal energy, biofuels, and nuclear energy, supplemented by energy-efficient management strategies. The exploration of renewable and green energy spans multiple aspects, including energy generation, conversion, storage, transportation, and utilization, driven by the pressing global challenge of curbing greenhouse gas emissions and their detrimental impact on our environment and health. Functional materials with novel properties play a pivotal role in this exploration.

Nanomaterials, distinguished by their nanometer-scale dimensions ranging from 1 nm to 100 nm, have come to the forefront, displaying superior properties compared to their bulk counterparts. Metal nanoparticles were employed by the Romans as early as the fourth century AD in the ancient glass industry, and later found applications in late-medieval European church windows [8]. The invention of the transmission electron microscope in 1931 [9] laid a solid foundation for investigations of nanomaterials, enabling direct observations of submicron and nanoscale materials [10]. The global wave of nanomaterial investigations took off with the discovery of buckminsterfullerene in 1985 [11], marking the advent of modern nanoscience and nanotechnology. The synthesis of carbon nanotubes in 1991 [12] further catalyzed this trend. The rediscovery of 2D graphene in 2004 [13] ignited a flourishing era in the field of nanoscience/nanotechnology. To date, a variety of nanomaterials, including 0D nanoparticles/quantum dots, 1D nanowires/tubes/fibers, 2D nanosheets, and their various combinations, as well as 3D bulk materials, have been extensively investigated in academia and industry. These materials range from inorganic metals, semiconductors, and insulators to organic nanomaterials such as cellulose [14].

Among their diverse applications, nanomaterials have been harnessed in renewable energy and environmental protection [15] due to their unique physicochemical properties, including electrical, magnetic, electronic, optical, thermal, mechanical, chemical, biological, and clinical properties. These distinctive properties are derived from their novel size-dependent physics and chemistry, driven by the strong confinement of electrons, photons, and phonons at the nanoscale within nanomaterials. To date, nanostructured materials have been investigated for advanced energy conversion [16], including thermoelectric devices [17], photovoltaic devices [18], and water splitting [19,20], and for electrochemical energy storage devices [21,22], such as supercapacitors [23,24], batteries [25,26], and fuel cells [27,28], as well as for various sensors like gas sensors [29,30], ion sensors [31,32], thermal sensors [33,34], and even protein sensors [35,36] and glucose sensors [37,38]. Their high surface/volume ratio and confinement properties make them particularly relevant for energy conversion and storage. Furthermore, nanomaterials are even employed in digital data storage with ultra-low energy consumption [39].

Among various electrochemical energy storage devices, supercapacitors have attracted significant attention due to their remarkable attributes, including high energy density, high power density, long cycle life, and rapid charge-discharge capabilities [40,41]. However, the current research on pseudocapacitive materials, which serve as supercapacitor electrode materials, faces challenges such as volume expansion, limited cycling performance, and high electrode resistance, which impose significant limitations on practical applications. To overcome these challenges, researchers have initiated the exploration of innovative electrode materials.

This Special Issue highlights several recent research breakthroughs that have transcended the limitations of conventional pseudocapacitive materials, showcasing the potential of novel electrode materials in supercapacitors. These advancements encompass N or P-doped porous MXene, N-doped carbon nanofiber-supported Fe_3_C/Fe_2_O_3_ nanoparticle composites, carbon nanosheet self-supported electrode materials utilizing aluminum foil substrates, and magnetic CuFe_2_O_4_ nanoparticles for supercapacitor applications. These research findings provide fresh insights and methodologies for the design and fabrication of high-performance supercapacitors, thus making substantial contributions to the advancement of energy storage technologies.

Pseudocapacitive materials currently utilized in supercapacitors face challenges such as volume expansion, limited cycling performance, and high electrode resistance [42,43]. To overcome these issues, researchers have begun to explore innovative electrode materials, including MXene, which boasts excellent conductivity, hydrophilicity, a large specific surface area, and tunable interlayer spacing. In this vein, Chen et al. made notable enhancements to the electrochemical performance of Ti_3_C_2_T*_x_* MXene electrodes through P doping, which widened the interlayer spacing of MXene, facilitating the diffusion of electrolyte ions and optimizing the surface electronic state, thereby enhancing conductivity. This modification led to a higher capacitance and improved cycling stability of P-doped MXene electrodes. Furthermore, Hu et al. successfully bolstered the electrochemical performance of MXene electrodes through hydrothermal treatment and alkaline solution processing. The treated MXene samples exhibited significantly increased gravimetric capacitance, reaching a peak value of 543 F g^-1^, approximately 250% higher than the original value. This improvement can be attributed to the expanded and homogenized interlayer spacing of MXene, providing ample space for electrolyte ion storage. The substitution of F-terminations with O-containing groups enhanced the hydrophilicity of MXene, facilitating the infiltration of electrolytes onto its surface and offering additional electrochemically active sites. Moreover, the treatment led to an increased oxidation state of titanium in MXene, rendering it more susceptible to reduction reactions. These findings provide valuable insights into the design and application of MXene materials, thereby holding significant implications for the development of high-performance supercapacitors and other electrochemical energy storage devices.

This Special Issue underscores several recent research breakthroughs that have surpassed the constraints of traditional pseudocapacitive materials, exhibiting the potential of novel electrode materials for supercapacitors. These advancements include N or P-doped porous MXene, N-doped carbon nanofiber-supported Fe_3_C/Fe_2_O_3_ nanoparticle composites, self-supported carbon nanosheet electrode materials that utilize aluminum foil substrates, and magnetic CuFe_2_O_4_ nanoparticles for supercapacitor applications. These research revelations offer fresh perspectives and methodologies for the design and fabrication of high-performance supercapacitors, thereby delivering significant contributions to the advancement of energy storage technologies.

The rise of flexible electronic devices has established the energy density of flexible energy storage devices as a critical factor restricting their application [44,45]. To address this challenge, Tao et al. [46] developed N-doped porous MXene (Ti_3_C_2_) as a self-supporting electrode material to boost the energy storage performance of flexible supercapacitors. This MXene material exhibited excellent conductivity and hydrophilicity, a large specific surface area, and adjustable interlayer spacing. Additionally, Li et al. studied N-doped carbon nanofiber-supported Fe_3_C/Fe_2_O_3_ nanoparticle composites, which displayed remarkable electrochemical performance in supercapacitors. These studies offered valuable insights into and contributions to the application of supercapacitors and the advancement of the energy storage field. Moreover, Zheng et al. [47] devised a method for fabricating self-supported carbon nanosheet electrode materials based on Al foil substrates, exploring the influence of reaction solution concentration on their structure and electrochemical performance. This straightforward and efficient method resulted in optimal samples with superior areal capacitance, demonstrating excellent rate performance and cycling stability. Furthermore, magnetic materials have garnered significant attention for energy storage applications. Liang et al. investigated the potential application of magnetic CuFe_2_O_4_ nanoparticles in energy storage. Their study revealed the exceptional pseudocapacitive characteristics of CuFe_2_O_4_ within a negative potential range, laying a foundation for further research on the utilization of magnetic/pseudocapacitive materials in energy storage. These studies provided fresh insights into the design and fabrication of supercapacitors, and contributed to the progress of the energy storage field. Further research and advancements are anticipated to yield even greater breakthroughs in these materials for the energy sector.

In the face of an intensifying global energy crisis, the quest for efficient and sustainable energy conversion and storage technologies has emerged as a primary focus of contemporary scientific research. However, the enhancement of these devices’ performance to meet the escalating energy demands remains an ongoing challenge. This Special Issue also showcases several trailblazing studies aiming to optimize the performance of electrochemical energy storage devices via innovative material design and processing methodologies. Firstly, Xie et al. put forth a report on the in situ growth of Mo-doped Cu_2_S nanosheets on a three-dimensional copper foam, functioning as an efficient electrocatalyst for a hydrogen evolution reaction. Their research furnished promising candidate materials for the development of novel energy-related and optoelectronic devices, offering valuable insights into the regulation of morphology and electronic structure for enhancing hydrogen evolution performance. Next, Wang et al. proposed a photothermal layer based on flower-like carbon nanoparticles for effective solar-driven interfacial evaporation, holding potential applications in water treatment. This study presented a novel strategy for the utilization of carbon nanomaterials in water purification endeavors. Furthermore, we spotlighted the innovative research in polymer design by Soroceanu et al. They synthesized a series of polymers incorporating the electron donor triphenylamine or carbazole and the electron acceptor naphthalene diimide. These polymers exhibited exceptional thermal stability and solubility while effectively facilitating electron and hole transport, laying the groundwork for the evolution of novel energy-related and optoelectronic devices. Lastly, Madondo et al. investigated the impact of electrode spacing on the performance of microbial fuel cells (MFCs). Their research findings offered invaluable guidance and insights for the advancement of high-performance MFCs within bioelectrochemical systems. Additionally, magnetic refrigeration technology, an innovative cooling approach based on the magnetocaloric effect, has attracted extensive attention in recent years. The evolution of this groundbreaking technology presents novel possibilities for the refrigeration industry, although it still faces various challenges in practical applications. Li et al. conducted a comprehensive study on the magnetic and mechanical properties of europium monosulfide (EuS). They procured EuS powder through the sulfurization of europium oxide powder and subsequently sintered EuS under various temperature conditions using spark plasma sintering. Their research provided valuable guidance and references for effectively enhancing the magnetic and mechanical properties of EuS through the regulation of sintering temperature and doping elements, thereby progressing the development of magnetic refrigeration technology.

In addition to conventional energy conversion/storage techniques, this Special Issue also features two specialized energy storage materials/devices, specifically phase-change materials and magnetic tunnel junctions. These two comprehensive reviews broadened the applications of nanomaterials in the ecosystem of energy conversion and storage.

Magnetic tunnel junctions (MTJs), composed of two-dimensional insulating nanosheets sandwiched between two magnetic layers, have been extensively utilized in magnetic memory [48,49] and logic gates [50] due to their tunneling magnetoresistance. These devices have also been harnessed to harvest heat [51,52], solar energy [53,54], and mechanical energy [55,56], and are used as capacitors [57] and batteries [58] for energy storage. However, MTJs face crucial challenges regarding their performance and reliability. In this context, this editorial provides a comprehensive analysis and exploration of MTJs under irradiation conditions. Seifu et al. [59] conducted an in-depth review of MTJs based on MgO and their performance under different electromagnetic radiation environments, evaluating their radiation tolerance. The study examined the mechanisms of MgO tunneling layers, magnetic layers, and interfaces under radiation exposure extensively, offering critical theoretical insights into the origin of their radiation tolerance. This work provides readers with comprehensive information and holds significant reference value for understanding the performance and applications of nanomaterials/devices in radiation environments.

Latent heat is either absorbed or released during a phase transition. For instance, when ice melts, it absorbs 333.55 J/g of heat, and when water freezes into ice, it releases the same amount of heat. When a phase-change material (PCM) undergoes a transition between solid and liquid states, or when its internal structure changes, it either absorbs or releases significantly more energy compared to sensible heat storage. As a result, PCMs are invaluable for energy storage. To date, PCMs have found extensive applications in latent heat thermal-storage systems for heat pumps, solar engineering, spacecraft thermal control, and building applications [60,61]. In this Special Issue, Pereira et al. [62] provide a comprehensive overview of the applications of nanoscale phase-change materials in energy harvesting and conversion. The review delves into the major thermophysical properties of nanoscale phase-change materials and discusses their applications in solar thermal energy storage systems and photovoltaic-nanoscale phase-change materials systems. This work offers crucial guidance for the future development of the research into phase-change materials.

In conclusion, this Special Issue presents a comprehensive overview of the latest research on nanomaterials for energy conversion and storage. The highlighted studies illuminate the potential of novel electrode materials, the optimization of pseudocapacitive materials, and the exploration of flexible supercapacitors. These research findings make substantial contributions to the advancement of energy storage technologies and offer valuable insights for future developments in the field. As researchers continue to explore innovative approaches and address the challenges faced by these materials, we can anticipate even greater breakthroughs in the energy sector. The collective efforts showcased in this Special Issue demonstrate the commitment of scientists to finding sustainable solutions for the ever-growing energy demand.
